# 中药降解寡糖制备和结构表征的研究进展

**DOI:** 10.3724/SP.J.1123.2025.08024

**Published:** 2026-08-08

**Authors:** Junyi LIU, Yi ZHENG, Jingyu YAN

**Affiliations:** 1. 赣江中药创新中心，江西 南昌 330100; 1. Ganjiang Chinese Medicine Innovation Center，Nanchang 330100，China; 2. 植物化学与天然药物全国重点实验室，中国科学院大连化学物理研究所，辽宁 大连 116023; 2. State Key Laboratory of Phytochemistry and Natural Medicines，Dalian Institute of Chemical Physics，Chinese Academy of Sciences，Dalian 116023，China

**Keywords:** 中药多糖, 降解寡糖, 结构表征, 分离纯化, 结构-活性关系, **t**raditional Chinese medicine polysaccharides, degradated oligosaccharide, structural characterization, separation and purification, structure-activity relationship

## Abstract

中药多糖因具有免疫调节、抗肿瘤等优异生物活性而广受关注，但多糖存在分子质量大、单糖组成复杂、支链结构多样的问题，导致其生物利用度偏低、构效关系难以解析，进而限制了其应用。而寡糖分子质量低、结构相对简单且易于纯化与解析，成为揭示中药多糖构效关系的理想模型，其制备、分离纯化及结构表征是该领域的研究核心。本文以中药降解寡糖为核心研究对象，首先从降解策略层面，系统探讨了化学水解、氧化降解、生物酶解及辅助降解等方法，对比分析了不同方法的降解机理、产物分布特征及适用范围，同时阐述了各类方法对寡糖聚合度、端基及支链结构的改造效应，以及对其生物活性的潜在调控作用；随后针对降解寡糖常用的分离纯化色谱技术（凝胶过滤色谱、离子交换色谱、亲水作用色谱及多孔石墨化炭色谱）展开系统评述，详细介绍了各类色谱技术的分离原理、适用场景及在降解寡糖分离中的具体应用；最后针对单糖组成分析、甲基化分析及核磁共振波谱等寡糖结构表征技术，阐明了不同技术间的互补性与各自的局限性。相信随着中药降解寡糖在分离纯化与结构解析领域的持续突破，以及人工智能与多组学等新技术的深度融合，其构效关系将日趋明朗，在功能性食品开发、新药研发及成果转化领域也将发挥更为重要的作用。

## Abstract (en)

Polysaccharides derived from traditional Chinese medicine （TCM） have attracted widespread attention due to their excellent bioactivities， such as immunomodulatory， anti-inflammatory， and anti-tumor effects. However， these polysaccharides exhibit inherent characteristics including large molecular weights， complex monosaccharide compositions， and diverse branched structures. These features result in low bioavailability and difficulties in unraveling their structure-activity relationships （SARs）， thereby imposing significant limitations on their practical applications. In contrast， oligosaccharides possess distinct advantages： they have low molecular weights， relatively simple structures， and are easy to purify and characterize structurally. Owing to these properties， oligosaccharides serve as ideal models for deciphering the SARs of TCM polysaccharides. Consequently， the preparation， separation and purification， and structural characterization of TCM oligosaccharides have become the core research focuses in this field.This paper firstly reviews various degradation strategies from the perspective of degradation approaches. These strategies include chemical hydrolysis， oxidative degradation， enzymatic hydrolysis， and assisted degradation methods. For each method， a comparative analysis of their degradation mechanisms， product distribution characteristics， and applicable scopes is conducted. Meanwhile， it elaborates on the modification effects of different methods on the degree of polymerization （DP）， anomeric configuration， and branched structure of oligosaccharides， as well as their potential regulatory roles in the bioactivities of oligosaccharides. Subsequently， this paper carries out a systematic review on the chromatographic techniques commonly used for the separation and purification of degraded oligosaccharides， including gel filtration chromatography （GFC）， ion-exchange chromatography （IEC）， hydrophilic interaction chromatography （HILIC）， and porous graphitized carbon chromatography （PGC）. For each chromatographic technique， this paper provides a detailed account of its separation principle， applicable scenarios， and specific applications in the separation of degraded oligosaccharides. Finally， regarding the oligosaccharide structural characterization techniques-such as monosaccharide composition analysis， methylation analysis， and nuclear magnetic resonance （NMR） spectroscopy-the paper clarifies the complementarity between different techniques and their respective limitations.In summary， this paper focuses on the application of combined strategies-such as chemical hydrolysis， enzymatic hydrolysis， and physicochemical hybrid methods—in the preparation of degraded oligosaccharides derived from TCM， as well as the roles and key points of separation techniques and characterization techniques in structural elucidation. Looking ahead， the preparation， separation and purification， and structural analysis of TCM oligosaccharides require the further establishment of standardized protocols. Additionally， the integrated application of artificial intelligence and multi-omics technologies should be actively promoted in this research field. It is believed that with the continuous in-depth exploration of degraded oligosaccharides， their SARs will become increasingly explicit. It is anticipated that progress in the purification and structural elucidation of TCM-degraded oligosaccharides， together with the convergence of artificial intelligence and multi-omics approaches， will bring clarity to their structure-activity relationships. This will， in turn， enable their more precise and impactful application in functional food design and pharmaceutical development.

中药多糖作为中医药体系中的重要活性成分，近年来因其在免疫调节^［1］^、抗肿瘤^［2］^、抗氧化^［3］^、抗病毒^［4］^及抗衰老^［5］^等多方面的生物活性而受到广泛关注，并且研究表明其生物活性与分子结构的高度复杂性和异质性密切相关^［6］^。中药多糖通常具有高分子质量、复杂的单糖组成及高度分支的结构特征^［7］^，这些结构特性在赋予其多样化生物功能的同时，也常导致溶解性差、生物利用度低及体内代谢复杂等问题，成为其应用的限制因素^［8，9］^。

大量实验证明多糖复杂的结构特征与其生物活性密切相关。Wang等^［10］^在绿茶中分离了一种由*β*（1→3）和*β*（1→6）连接的半乳糖构成主链、*α*（1→5）连接的阿拉伯糖构成支链的复杂结构多糖，具有显著的胰岛素分泌促进活性。但仅保留主链或支链结构均丧失活性，说明主链和支链的共同作用是其发挥活性的关键。Demleitner等^［11］^发现*β*（1→3）连接的葡聚糖具有明显的抗肿瘤活性，而由33%的*β*（1→3）和66%的*β*（1→4）连接的混合葡聚糖抗肿瘤活性大幅降低。Kraus等^［12］^证明了*β*（1→3）或*β*（1→6）葡萄糖连接的主链的“低分支状态”是其维持高抗肿瘤活性的关键结构特征。Zhu等^［13］^对半乳寡糖（galacto-oligosaccharides， GOS）和果寡糖（fructo-oligosaccharides， FOS）两类不同的非消化性寡糖进行功能对比，发现在相同条件下GOS表现出比FOS更强的抗菌作用。Cui等^［14］^证明了具有三螺旋立体结构的马尾松多糖抗病毒活性比较强。由此可见，糖链的精细结构对其生物活性起着决定性的作用。

近年来，降解生成的寡糖逐渐成为重要的研究方向，将多糖降解为寡糖是一种有效的结构改性策略，能够提升其理化性质与生物活性。寡糖是由2~20个单糖单位组成的小分子糖链^［15］^，其分子质量较小且结构相对简单^［16］^，适合用作生物活性研究的对象。降解寡糖的制备方法多样，最常见的方法主要包括酸催化水解和酶催化降解等^［17］^，能够有效获得具有特定生物活性的寡糖片段，为深入探讨中药多糖的生物活性提供了新的视角。

高效液相色谱（high performance liquid chromatography， HPLC）、核磁共振（nuclear magnetic resonance， NMR）和质谱（mass spectrometry， MS）等现代分析技术，使研究人员能够对降解寡糖的结构进行精细解析^［18］^。这些技术的运用不仅为理解不同寡糖之间的结构差异提供了重要依据，也为揭示中药多糖的生物活性与其分子结构之间的关系奠定了基础^［19］^。值得注意的是，寡糖的结构和组成对其生物功能具有深远影响。随着对降解寡糖生物活性机制的深入研究，越来越多的证据表明，寡糖片段不仅在传统医学中有着广泛应用，也在功能性食品和新药研发中展现出巨大潜力^［20］^。因此，系统研究降解寡糖的构效关系已成为推动中药多糖现代化应用的重要方向^［21］^。

综上所述，中药多糖的重要性及潜在生物活性使其成为研究热点，而复杂的结构性对研究提出了挑战^［22］^。寡糖的分子质量较低，结构相对简单，是多糖的组成片段和活性的基本单元，因此将多糖降解为寡糖片段，研究寡糖的活性机制是解析多糖发挥功能的策略之一。本综述旨在全面探讨降解寡糖的制备方法、分离纯化及结构解析技术的研究进展，以期为未来的研究提供参考信息、方法学启示和数据支撑，并对其前景进行了展望。

## 1 寡糖降解方法

### 1.1 化学水解法

#### 1.1.1 酸水解法

酸水解法是一种传统且应用广泛的多糖降解技术，其核心机理依赖于质子对糖苷键中氧原子的亲电攻击，最终导致糖苷键断裂^［23］^。该方法因操作简便，成本较低，在大规模预处理中仍具有实用价值。在实际应用中，针对不同结构的多糖和分析目的，需谨慎选择酸的种类与水解条件。

硫酸是早期常用的无机强酸，只有在一些特定类型的多糖水解中有应用，适用于某些含硫酸基团的多糖的初步降解。例如杨波等^［24］^将*ι*-卡拉胶（一种天然硫酸多糖）用0.1 mol/L的硫酸于60 ℃酸水解至聚合度（degree of polymerization， DP）2~20。张媛媛等^［25］^通过正交试验对附子多糖在硫酸中的降解进行研究，结果表明附子多糖在硫酸中的最佳降解工艺如下：酸浓度10%、温度100 ℃、时间2 h，此条件下寡糖含量可达36.17%。硫酸的高沸点和不挥发性导致反应后难以去除，通常需中和处理，会引入大量无机盐离子，干扰后续色谱、质谱的分析及纯化过程。

盐酸是另一种常用的强酸，吴琼等^［26］^采用盐酸降解法对银耳多糖进行降解，并发现低分子质量银耳多糖的分子质量在2~10 kDa范围内，活性高于未降解银耳多糖。徐强等^［27］^通过正交试验优化了盐酸水解琼胶的实验条件，并得到了琼胶寡糖。水解产物经乙醇沉淀、超滤除去大分子后，经凝胶柱层析可得到琼胶低聚糖各寡糖组分。盐酸水解多糖容易产生氯离子残留问题，可能严重干扰离子色谱分析和质谱检测，导致离子抑制或基线噪声，影响寡糖定性与定量的准确性。

三氟乙酸（trifluoroacetic acid， TFA）是实验室规模制备寡糖的最常用酸试剂。TFA的核心优势在于其低沸点和易挥发性，反应后可通过旋转蒸发或冷冻干燥去除，有效避免了无机盐离子的引入，极大简化了后续纯化流程并减少了对色谱-质谱分析的干扰。通过系统优化TFA的浓度、温度和时间，可在一定程度上控制水解偏向性，获得特定聚合度范围的寡糖片段。杜宝香等^［28］^采用1.5 mol/L TFA于80 ℃下降解北沙参多糖4 h，获得DP 4~11的葡寡糖；石丽霞等^［29］^在相似条件下（1.0 mol/L TFA， 80 ℃， 1 h）从黄芪多糖中得到具有免疫活性的DP9~19寡糖混合物。然而，即便使用TFA，酸水解固有的局限性依然存在。其反应仍是一个可能包含降解、脱水、重排在内的过程。在高效断裂糖苷键的同时，高温和质子也会引发显著的副反应，导致单糖进一步转化为多种副产物，如5-羟甲基糠醛（5-HMF）、糠醛及5-甲基-2-呋喃甲醛等。卢键梅等^［30］^的研究表明，pH、温度、金属离子等因素可促使葡萄糖和果糖转化为3-脱氧葡萄糖醛酮和5-HMF等杂质，3-脱氧葡萄糖醛酮会影响抗氧化活性^［31］^和抗炎活性^［32］^等，5-HMF会干扰抗氧化活性和免疫活性^［33］^等。所以这些副产物不仅降低目标寡糖回收率，更可能干扰后续的活性测试及定性与定量分析，最终影响产物的活性表征。

此外，质子对糖链的随机攻击本质决定了其水解过程难以精确控制，容易形成不同DP寡糖的复杂混合物，这种显著的异质性增加了后续分离纯化与结构解析的难度。因此，通过系统优化（如正交试验、响应面法）寻找针对特定多糖的“最佳”反应窗口，以提升目标DP范围寡糖得率并降低副反应，仍是酸水解研究的关键环节。

#### 1.1.2 氧化降解法

除了传统的酸水解，氧化降解法作为一种通过活性氧物种断裂糖苷键的策略，因其反应条件相对温和、环境友好而受到广泛关注^［34］^。该类方法的核心机制是利用氧化剂产生的自由基（如·OH）或活性中间体，对糖环或糖苷键进行选择性或非选择性攻击，从而实现多糖链的断裂。

过氧化氢（H₂O₂）降解法因成本低廉、后处理简单（分解产物为水）已成为该领域研究中应用最广泛的技术之一。例如，谭诗敏等^［35］^利用过氧化氢-抗坏血酸（H_2_O_2_-Vc）体系降解香水莲花多糖，成功获得了分子质量分别为129、46和15 kDa的3种组分，并发现降解后多糖的抗氧化活性显著提升。臭氧（O₃）降解法也是一种颇具前景的技术，臭氧分子本身及其在水溶液中分解产生的羟基自由基均可攻击多糖链。娄广庆等^［36］^使用臭氧对魔芋葡甘露聚糖进行降解，通过单因素试验得到最优反应条件分别是反应时间1 h、臭氧投加量6 g/h、料液比1%（g/mL）以及反应温度55 ℃，且降解的产物没有发生明显的基团变化，说明臭氧氧化在降低分子质量的同时，能较好地保留多糖的原始结构骨架，这对于维持其生物活性至关重要。高碘酸盐氧化法能特异性识别并断裂相邻羟基之间的C-C键，生成醛基，从而实现糖环的开环和糖链的切断，这种特性使其非常适用于多糖的结构解析和特定寡糖的制备。Pandeirada等^［37］^通过高碘酸氧化法，在温和（室温）或高温（70 ℃）条件下成功将桦木、瓜尔豆、罗望子种子、柠檬和小麦这5种植物多糖降解为寡糖，并通过二级质谱（MS/MS）推测部分结构单元。更有研究将高碘酸盐氧化与其他方法联用，以开发功能更优异的衍生化产物，de Carvalho等^［38］^用“高碘酸+次氯酸钠”两步法把海藻硫酸化多糖氧化成多羧基寡糖，并发现其抗凝血活性显著增强，这揭示了氧化降解不仅是降低分子质量的手段，更是定向修饰多糖结构、创造新功能的有效工具。

### 1.2 生物酶解法

生物酶解法是利用酶的特异性催化作用降解多糖以获得目标寡糖片段的关键技术。与化学降解法相比，酶解法具有条件温和、特异性强、副产物少等显著优势^［39］^，能够最大限度地保持糖链的结构完整性，获得DP分布集中、结构明确的寡糖产物^［40］^。

根据作用机制的不同，多糖降解酶^［41］^主要分为内切糖苷酶和外切糖苷酶两大类。内切糖苷酶能特异性识别并切割多糖链内部特定类型的糖苷键，其切割位点可位于该特定键型所在的任何位置，从而将长链多糖分割成不同DP的寡糖片段，是制备不同DP寡糖混合物的主要工具酶^［42］^。而外切糖苷酶则从多糖链的非还原末端依次切割，每次释放1~2个单糖单位^［43］^。

相较于化学降解法的宽泛DP分布，酶法产物更具均一性^［44］^，这对于获得结构与活性关系更明确的寡糖组分至关重要。在具体应用方面，内切糖苷酶展现出显著的价值。Wang等^［45］^的研究表明，人参鼠李半乳糖醛酸聚糖I（RG-I）的长寿与抗氧化活性主要依赖于其阿拉伯糖侧链。他们通过内切-1，5-*α*-阿拉伯聚糖酶特异性去除该侧链后，核心骨架的上述活性几乎完全丧失，证明该侧链结构是发挥人参果胶长寿活性的关键结构。刘振锋^［46］^以蟹壳为原料，经粉碎、灭菌后，先进行复合菌发酵，再采用由短柄帚霉-红球菌发酵物及纤维素酶组成的复合内切酶制剂进行协同酶解，最终成功制备出壳寡糖；Zhang等^［47］^则通过*Bacillus* sp. MSJ-5分泌的内切-*β*-甘露聚糖酶将魔芋粉水解成DP 2~6的甘露低聚糖。这些研究表明，内切糖苷酶能够通过选择特定的酶制剂，实现对多糖链的精准切割，获得结构明确和DP分布更窄的寡糖产物。

### 1.3 辅助降解法

辅助降解法也是近年来在多糖降解领域逐渐受到重视的研究策略之一。通常将物理手段（如超声波、微波、机械力等）与化学或酶催化降解相结合，通过协同效应优化反应条件，从而提高降解效率、提升目标产物的选择性与产率。具体研究中，低共熔溶剂（deep eutectic solvent， DES）通过脱除木质素、保护酶活性显著提升中药多糖降解效率。例如解先利等^［48］^采用氯化胆碱-乙醇胺DES预处理甘草渣，使酶解木制纤维素效率提升1.58倍。Chen等^［49］^提取了合欢皮和黄茅的纤维素，并采用DES将纤维素分解为可溶性低聚纤维素糖。但DES体系引入的溶剂去除与后处理步骤增加了工艺复杂性，且需要系统优化影响产物纯度与功能性的变量^［50］^。胡皓程等^［51］^将H_2_O_2_氧化水解与超声物理水解联用，对黑木耳粗多糖进行处理，经过阴离子交换柱层析与凝胶过滤柱层析分离，获得黑木耳寡糖产物。Zhou等^［52］^则将酸性木聚糖酶与超声波结合进行酶水解，以提高玉米芯麸皮中木寡糖（xylooligosaccharides， XOS）的产量。总体而言，物理-化学联用法通过多途径协同作用，为多糖降解、寡糖产物的产量与结构表征以及潜在的生物活性研究提供了新的技术平台与应用前景。上述4种主要方法的关键特性对比汇总如表1所示。

**表1:** 中药多糖主要降解方法对比

Comparison of degradation methods	Acid hydrolysis	Oxidative degradation	Enzymatic hydrolysis	Assisted degradation
Core mechanism	electrophilic attack of H⁺ on the glycosidic oxygen atom	non-specific attack by ROS （e.g.， ·OH） on glycosidic bonds and sugar rings	highly specific recognition and cleavage of target glycosidic bonds	enhancement of mass transfer and reaction efficiency via physical fields or novel solvents
Specificity	non-specific cleavage of all acid-labile bonds	non-specific	highly specific to certain linkage types and sugar units	inherits the specificity of the primary degradation method
Condition mildness	high temperature and strong acid required	relatively mild； special equipment for ozone	typically under mild conditions	can lower the required temperature or time of the core reaction
Product controllability	mixture with a broad DP range， low controllability	preliminary control of degradation extent	can directionally produce oligosaccharides of specific DP	significantly improves the controllability of the core method
Main by-products/effects	5-HMF， furfural， monosaccharides， monosaccharide degradation	uronic acid loss； sugar ring opening； aldehyde/carboxyl group introduction	few side reactions， pure products	generally reduced； milder conditions reduce secondary degradation
Structural destructiveness	dehydration； configuration change due to harsh conditions	hydroxyl oxidation； C-C bond cleavage	perfectly preserves sugar ring structures and configurations	aims to mitigate the destructiveness of the core method
Environmental friendliness	strong acid usage； waste acid streams	H₂O₂ decomposes to H₂O； expensive periodate	green process， mild conditions， no hazardous waste	improved efficiency leads to greener processes
Typical application	initial depolymerization， large-scale pretreatment， sample prep for analysis	producing antioxidant oligosaccharides， structural sequencing， material modification	directed preparation of well-defined oligosaccharides for detailed SAR studies	enhanced efficiency of traditional methods

ROS： reactive oxygen species； DP： degree of polymerization； HMF： hydroxymethylfurfural； SAR： structure-activity relationship； DES： deep eutectic solvent.

## 2 寡糖单体的分离纯化

纯化寡糖单体通常依赖多种色谱技术，根据分离原理的不同，这些技术主要可分为凝胶过滤色谱、离子交换色谱、亲水作用色谱和多孔石墨化碳色谱等，下文将具体详述各个技术优缺点。

### 2.1 凝胶过滤色谱

凝胶过滤色谱（gel permeation chromatography， GPC），也被称为尺寸排阻色谱（size exclusion chromatography， SEC）是依据分子在水相或有机相溶液中的流体力学体积（或尺寸）差异进行分离的一种关键技术，其核心机理为尺寸排阻效应：较大分子因无法进入固定相填料孔隙而沿填料间隙快速流过色谱柱，被较早洗脱；较小分子则可进入孔隙内部，流径更长，保留时间也更长，从而实现按分子尺寸从大到小的分离^［53］^。这一分离特性使其成为寡糖研究中按照聚合度进行分级制备的重要工具，常用于对复杂的水解产物进行初步分组，去除体系中残留的未降解大分子多糖或盐类等小分子杂质，并可基于已知分子质量的标准品绘制曲线，对寡糖组分的表观分子质量分布进行初步评估。然而，GPC的应用也存在明显的局限性。其分离分辨率受限于填料的孔径分布，对于分子质量极为接近的组分或同一分子质量下不同结构的异构体区分能力有限^［54］^。

尽管如此，GPC凭借其条件温和、不依赖样品电荷、能与多种检测器（如示差折光检测器RI、多角度激光光散射仪MALLS）在线联用的优势，实现分子质量的相对/绝对定量，并辅助判断寡糖在不同DP区间的分布；但分离结果易受柱内吸附、样品降解及溶解度等因素影响，因此需对标准物质标定、线性范围验证提高结果的可靠性^［55］^。郝林华等^［56］^通过水提醇沉提取出牛蒡中粗糖组分，并用SephadexG-50凝胶柱层析分离纯化，得到水溶性的牛蒡寡糖。Tsai等^［57］^通过Smith降解法得到灵芝多糖，对其进行酸水解，使用Biogel-P10凝胶柱对灵芝多糖酸解馏分进行分离纯化，得到灵芝寡糖组分。这些应用案例体现了该方法在从多糖粗提物中按尺寸快速捕获活性寡糖片段方面的实用价值。

### 2.2 离子交换色谱

离子交换色谱（ion-exchange chromatography， IEC）作为最早期的色谱技术之一，至今仍是分离纯化蛋白质、核酸及糖类等生物大分子的常用手段。其分离基础依赖于目标物与固定相之间可逆的离子相互作用，通过样品离子在流动相与固定相间的动态平衡实现分离^［58］^。根据不同的离子模式可分为阴离子交换色谱和阳离子交换色谱。IEC对带有电荷的寡糖（如含有羧基、磺酸基等负电荷或正电荷的寡糖）表现出卓越的分离能力，可根据电荷密度、取代基位置等差异实现有效分离^［59］^。

IEC的应用范围不仅限于自身带电的分子。现代高效阴离子交换色谱（HPAEC）的一项重大突破在于，其采用高pH值的碱性淋洗液（如NaOH），使中性寡糖分子上的羟基（-OH）发生“原位电离”，生成带负电的氧阴离子（-O⁻），从而能够与固定相发生作用。这一机理巧妙地将离子交换的应用拓展至中性糖领域，使其成为分离分析中性寡糖及其同分异构体的权威方法。Kazłowski等^［60］^通过CarboPac（TM）PA100高效阴离子交换色谱柱分离纯化了琼胶寡糖。李仁勇等^［61］^使用NaOH淋洗液淋洗，通过CarboPac PA10高效阴离子交换柱结合脉冲安培检测器充分分离了7种寡糖类成分。IEC在揭示寡糖电荷分布和修饰状态方面具有独特优势，常与其他分离与表征手段联合使用，以获得更全面的结构信息。需要注意的是，离子强度、pH值及洗脱体系等参数对分离效果有显著影响^［62］^，实际应用中往往需要结合多种表征方法来确证结果的可靠性。

### 2.3 亲水作用色谱

亲水作用色谱（hydrophilic interaction liquid chromatography， HILIC）通过极性固定相和高比例有机相形成的独特微环境实现分离，其保留机制源于溶质在固定相表面富水层与流动相之间的分配及其亲水性差异，这一特性使其成为分离强极性化合物，尤其是中性及极性寡糖的理想选择^［63］^。与反相色谱（reverse phase liquid chromatography， RPLC）互补，HILIC的核心优势在于其对糖类细微结构差异的高分辨能力。它不仅可有效按DP分离寡糖，更能区分连接位点、异头构型（*α*/*β*）和序列不同的同分异构体^［64］^。这一优势使其与质谱联用成为糖类精细结构解析的强有力工具。李亚栋等^［65］^使用亲水色谱-高效液相色谱-蒸发光散射检测器（HILIC-HPLC-ELSD）构建了炙黄芪多糖酶解产物指纹图谱。吴岩斌^［66］^采用亲水作用色谱/超高效液相色谱-高分辨质谱（HILIC/UHPLC-HRMS）法建立金线莲及其混伪品多糖部分酸水解的特征寡糖图谱。然而，HILIC分离性能的优化是一项挑战，其对有机相比例、柱温、流动相pH值、缓冲盐浓度等参数极为敏感，微小的变化可能导致保留时间和选择性的显著改变，方法的稳健性和重现性需通过系统优化与严格控制来保证。

### 2.4 多孔石墨化碳色谱

在众多色谱技术中，多孔石墨化碳色谱（porous graphitized carbon， PGC）凭借其独特的多孔石墨化碳固定相，在糖类同分异构体的分离方面展现出近乎无可比拟的高分辨率。其分离机制超越了简单的反相色谱的分配原理，同时包含疏水作用、电子相互作用（*π*-*π*和*n*-*π*）以及空间位阻效应，从而能敏锐地区分由异头碳构型（*α*/*β*）、糖苷键连接位点，甚至单糖取代基差异所导致的微小空间结构变化，使其成为解析糖类精细结构的首选^［67］^。

PGC的优势在于其卓越的异构体分辨能力，Robinson等^［68］^通过PGC-MS联用技术在小麦茎中分离出DP 2~20的寡糖。Li等^［69］^对鹿茸菇多糖进行分离纯化，获得均质组分后，并对其进行酸水解；最终利用PGC-MS联用技术对水解产物进行分析，成功鉴定出系列不同分子质量的寡糖片段。

然而，PGC的强大能力也伴随着独特的挑战。其中最值得注意的是“双峰现象”，即一种寡糖可能在色谱图中出现两个峰，这通常源于寡糖还原端*α*/*β*构型异构体被有效分离而产生的，对于含有多种寡糖的混合物，构型异构体在谱图上会交织出现，增加了谱图复杂程度，为样品分析带来了较大挑战，这一情况可在超高柱温条件下予以缓解^［70］^。此外，PGC固定相对某些结构具有极强的保留能力，要求方法开发时需精心优化梯度洗脱条件（如有机相比例、添加改性剂），以避免严重的峰拖尾^［71］^。以上不同的分离纯化技术的优缺点及适用性如表2所示。

**表2:** 不同分离纯化技术的适用性总结

Chromatography technique	Stationary phase	Main advantages	Typical application scenarios	Application cases	Ref.
GPC/SEC	porous particles	enables separation by molecular size/hydrodynamic volume； mild conditions	fractionation by degree of polymerization； desalting； removal of large/small molecules	isolation and purification of oligosaccharides produced by hydrolysis of oat *β*-glucan	［^72^］
IEC	charged functional groups （e.g.， DEAE， CM）	high capacity for charged molecules； achieves separation by charge density/properties	separation of acidic/charged oligosaccharides； often serves as the first purification step	separation of hydrolyzed oligosaccharides from *Plantago major* L.	［^73^］
HILIC	polar groups	exhibits superior performance for polar compounds； high resolution for isomers	separation of neutral/ polar oligosaccharides and isomers； often coupled with MS	separation of native cello-oligosaccharides and oxidized cello-oligosaccharides produced by cellulose hydrolysis	［^74^］
PGC	porous graphitized carbon	exceptional resolution for isomers； maintains stability under wide pH range	separation of challenging isomeric oligosaccharides for detailed structural analysis	separation and analysis of oligosaccharides from multiple sources	［^75^］

GPC： gel permeation chromatography； SEC： size exclusion chromatography； IEC： ion-exchange chromatography； HILIC： hydrophilic interaction liquid chromatography； PGC： porous graphitized carbon.

## 3 结构表征

在纯化得到的寡糖单体的结构解析阶段，通常通过单糖组成分析、质谱技术、甲基化分析和核磁共振技术4种互补方法来确定结构。这4种技术的分析范围、优势与局限如表3所示，它们共同构成了一个完整的结构解析寡糖体系。

**表3:** 寡糖结构解析4种关键技术对比表

Technique	Primary analytical scope	Core advantages	Major limitations
Monosaccharide composition analysis	qualitative/quantitative analysis of monosaccharide types and molar ratios	high sensitivity； provides fundamental primary information for structural elucidation	prone to monosaccharide degradation during acid hydrolysis； no glycosidic linkage/sequence information
MS	molecular weight determination； molecular formula inference； sequence/partial linkage elucidation	extremely high sensitivity， minimal sample consumption； detailed fragment ion information； compatibility with LC/GC for analyzingcomplex mixtures	limited distinction of isomeric monosaccharides； difficult linkage differentiation via isobaric fragments； no *α*/*β* anomeric configuration information； quantitative accuracy dependent on ionization efficiency
Methylation analysis	glycosidic linkage position/branching point identification； hydroxyl group involvement in linkages	recognized as authoritative for direct linkage determination； clear branching architecture revelation； intuitive results comparable with standard libraries	complex； lengthy； multi-step derivatization； ineffective for acidic/sulfate-containing oligosaccharides； destructive to analytes； no sequence/anomeric configuration information
NMR spectroscopy	complete molecular structure elucidation； *α/β* anomeric configuration； sugar ring conformation； linkage sequence； branch point location	provides complete solution-phase stereochemical and conformational information	requires high-purity， milligram-scale samples； complex spectrum interpretation due to severe signal overlap

### 3.1 单糖组成分析

在寡糖及多糖结构解析的起始阶段，单糖组成分析通过对寡糖经酸水解或酶解后的单糖定性与定量^［76］^，当下常用方法包括两类：一是衍生化法，通过将单糖转化为易分离/易检测的衍生物（如1-苯基-3-甲基-5-吡唑啉酮衍生化法），通过紫外检测器做定性定量；二是直接检测法，利用高效离子交换色谱-脉冲安培检测（HPAEC-PAD）或其他检测器（如示差折光检测器、电雾式检测器）直接对游离单糖进行分离与定量^［77］^。但需警惕水解过程对某些糖的降解或结构损失，以及某些糖无法衍生化（如果糖），且在色谱上色谱峰容易重叠。

### 3.2 质谱技术

质谱在寡糖结构解析中提供分子质量与碎片信息的直接证据，通过获得完整分子式、分子质量分布，以及特定碎片模式，能指向可能的端基与连接位点的线索，帮助排除部分同分异构体并给出候选序列^［78］^。然而，植物寡糖在裂解过程中产生的B/Y型碎片离子，其质荷比（*m/z*）可能极为相近甚至完全相同。例如，*α*（1→4）连接的葡萄糖和*β*（2→1）连接的果糖在单糖组成与连接方式上均不相同，但仅凭碎片质量数往往无法提供确凿证据^［79］^。且植物寡糖中最常见的己糖，如葡萄糖、半乳糖和甘露糖，其分子质量均为180 Da；而戊糖如阿拉伯糖和木糖分子质量均为150 Da。这些同分异构体在MS/MS中的碎片模式也高度相似，并且裂解主要发生在糖苷键和环醚键上，因此仍需要依赖其他方法辅助，最终确认结构。Guitteny等^［80］^使用芬顿反应氧化降解地衣多糖，并使用基质辅助激光解吸电离飞行时间质谱仪（matrix-assisted laser desorption ionization-time of flight mass spectrometry， MALDI-MS）检测产生的寡糖分布特征，检测到该降解方法在糖单元环上的还原端或非还原端产生环内裂解的副产物。此外，分子在离子化、传输和碰撞过程中会带有一定内能，这可能导致一些易断裂的基团发生脱落。例如，寡糖中硫酸基太多，易造成软电离过程中的脱落现象。杨波等^［24］^在对硫酸化地衣寡糖进行质谱分析时观察到，DP8~16的寡糖中均存在不同程度的脱硫酸基脱落。

### 3.3 甲基化分析

甲基化分析是揭示寡糖中糖苷键连接方式的关键方法之一。常用的实验操作是对寡糖进行完全甲基化，使游离羟基全部转化为甲基，然后经酸水解、还原、乙酸化得到甲基化的醇衍生物，通常称为部分甲基化的醛糖醇乙酸酯（partially methylated alditol acetates， PMAAs）。PMAAs的甲基化位点与保留的糖苷键共同决定其在气相色谱-质谱中的碎片模式与保留时间，从而推断出糖单元之间的连接位置以及分支点的结构信息^［81］^。甲基化分析能提供糖苷键连接位点的直接证据，然而，对于含酸性基团修饰的寡糖，或高分子质量、杂质较多的样品，该方法往往难以实现充分甲基化，从而影响后续位点推断的准确性。其次甲基化分析无法提供异头碳的构型信息（*α*-或*β*-连接），糖苷键的立体构型对其生物活性至关重要，但甲基化过程会破坏异头碳的手性中心，生成的PMAAs衍生物无法保留原始的异头构型信息。此外，甲基化分析无法提供糖链的序列信息^［82］^，甲基化分析可以提供每个糖环上有哪些位置被连接，但它是一种“破坏性”的分析，将整条糖链打碎成单个的修饰单糖。因此，它无法揭示这些不同连接位点的糖单元是以何种顺序排列成链的，也无法确定分支点在整个分子中的确切位置。

### 3.4 核磁共振技术

NMR是直接揭示糖单元之间连接关系与构型的关键工具。通过一维谱图（^1^H、^13^C）与二维谱图（如异核单量子相干谱（heteronuclear single quantum coherence，HSQC）、异核多键相关谱（heteronuclear multiple bond correlation，HMBC）、全相关谱（total correlation spectroscopy，TOCSY）等）可以定位糖单元位点、确定*α*/*β*构型、解析糖链的结构与分支情况，并在某些情况下给出糖单位的相对顺序信息^［83］^。获得高信噪比与良好分辨率通常需要较纯的样品和较高的样品浓度，且样品在D_2_O等溶剂中的溶解条件也会影响化学位移与耦合常数的解析。结合前3种技术的结果，NMR能提供最直接的连接关系与构象证据。然而，对于DP较高或结构复杂的寡糖，仅凭NMR直接解析其完整结构仍然面临巨大挑战，严重的信号重叠与狭窄的化学位移分布是首要难题。寡糖的质子（^1^H）化学位移主要集中在一个非常狭窄的区间（*δ* 3.0~4.4），特别是环上的大量H-2至H-6质子，其化学环境高度相似，导致信号严重堆积、难以分辨。尽管二维谱可以缓解这一问题，但随着糖链DP增加和分支变多，信号重叠会呈指数级加剧^［84］^。

综上所述，寡糖的结构解析是一项复杂的系统工程，除结构简单和DP低的寡糖以外，只靠单一技术很难能够提供其完整、准确的立体化学结构信息^［85］^。上述4种技术各具独特优势，但也均存在无法克服的固有局限性，它们之间构成了互补与印证关系。

## 4 结论与展望

中药现代化研究旨在借助现代科技手段，明确中药发挥疗效的物质基础和作用机制。中药多糖作为一种复杂的高分子化合物，其构效关系并不明确，而中药降解寡糖作为复杂结构多糖的组成片段，具有分子质量低、结构简单、易于纯化与解析等特点，是探索中药多糖生物活性机制的理想模型。

中药降解寡糖的研究过程包括降解、分离、纯化与结构表征。当前化学水解、酶解以及物理-化学联用的组合策略各有优势：酸水解操作简单，成本低，但易产生副产物，DP分布宽泛并可能破坏敏感结构；酶解条件温和，选择性更高，对目标片段的定向性利用潜力大，但常受底物特异性和酶种类的限制；物理-化学联用方法如微波辅助水解、热水-酶解级联、酶解-酸饱和化级联以及金属螯合剂协同酶解在提高水解效率与定向产物生成方面展现出显著潜力，逐步突破了单一策略的局限。分离与表征方面，GPC、IEC、HILIC、PGC等分离手段与单糖分析、质谱、甲基化分析、NMR等结构表征手段构成了较为完整的“分离-定性-定量-结构解析”体系，为构效关系研究提供了可靠支撑。然而，活性物质的定位仍存在模糊性，关键活性构象难以捕捉，对多靶点机制的阐释也仍具挑战性。在此背景下，针对不同中药及其多糖类型，选择合适的降解与分析策略，成为值得深入探索的问题。

在未来，可通过依托前沿技术与多学科交叉融合寻求突破。例如：1）利用人工智能（AI）的集成应用革新寡糖分离与表征的效率。在分离纯化环节，可利用机器学习算法，基于历史色谱数据（如固定相类型、流动相配比、洗脱梯度、目标寡糖保留时间）构建预测模型，自动优化针对特定结构寡糖（如酸性寡糖、中性寡糖同分异构体）的分离条件，规避传统“试错法”的烦琐流程；在结构表征环节，深度学习模型可赋能复杂谱图解析。例如，针对高聚合度寡糖的NMR信号重叠问题，AI可通过训练海量谱图数据，自动识别并拆分重叠质子信号；对于多级质谱（MS/MS）碎片解析，AI能快速匹配碎片离子与已知寡糖结构数据库，精准推断糖苷键连接方式与端基构型，大幅缩短人工解析周期。2）利用多学科技术的深度融合开展寡糖的构效关系研究，可尝试将生物信息学与代谢组学等学科结合，基于中药寡糖的结构特征（如聚合度、糖苷键类型、支链修饰），构建“寡糖结构-代谢通路-生物活性”的关联数据库，通过大数据分析与实验筛选快速锁定关键活性结构单元，避免传统“逐一结构验证”的低效模式。

近年来，中药寡糖在功能性食品领域得到广泛应用，且在新药研发领域展现出广阔的应用前景。尽管近年来降解寡糖研究取得了相当大的进展，但研究人员仍然面临着重大挑战。相信随着降解寡糖研究的不断深入，未来有望更清晰地明确关键结构单元及其作用机制，从而推动中药多糖在功能性食品与新药研发中的转化应用。
